# Transforming nephrology through artificial intelligence: a state-of-the-art roadmap for clinical integration

**DOI:** 10.1093/ckj/sfag004

**Published:** 2026-01-08

**Authors:** Wisit Cheungpasitporn, Ambarish Athavale, Lama Ghazi, Kianoush B Kashani, Tiago Colicchio, Jay L Koyner, Jin Chen, Joachim H Ix, Girish Nadkarni, Javier A Neyra

**Affiliations:** Division of Nephrology and Hypertension, Department of Medicine, Mayo Clinic, Rochester, MN, USA; Division of Nephrology, University of California, San Diego, La Jolla, CA, USA; Department of Medicine, University of Alabama at Birmingham, Birmingham, AL, USA; Division of Nephrology and Hypertension, Department of Medicine, Mayo Clinic, Rochester, MN, USA; Division of Pulmonary and Critical Care Medicine, Department of Medicine, Mayo Clinic, Rochester, MN, USA; Division of Nephrology and Hypertension, Department of Medicine, Mayo Clinic, Rochester, MN, USA; Section of Nephrology, Department of Medicine, University of Chicago, Chicago, IL, USA; Department of Medicine, University of Alabama at Birmingham, Birmingham, AL, USA; Division of Nephrology, University of California, San Diego, La Jolla, CA, USA; Division of Nephrology, Icahn School of Medicine at Mount Sinai, NY, NY, USA; Department of Medicine, University of Alabama at Birmingham, Birmingham, AL, USA

**Keywords:** artificial intelligence, acute kidney injury, chronic kidney disease, dialysis, kidney transplantation

## Abstract

Artificial intelligence (AI), encompassing machine learning, deep learning and generative AI, is poised to redefine nephrology by enabling earlier detection, more precise risk stratification and workflow-integrated clinical decision support across the spectrum of kidney disease. This state-of-the-art review synthesizes emerging applications of AI in acute kidney injury (AKI), chronic kidney disease (CKD), dialysis and kidney transplantation, with attention to clinical integration, real-world validation, workflow implementation and translational challenges. In AKI, predictive models trained on high-frequency electronic health record data and intensive care unit telemetry have demonstrated strong performance in forecasting critical events, yet translation into routine clinical workflows remains limited. In CKD, machine learning tools support progression risk stratification and phenotype clustering, with the potential to inform individualized surveillance and therapy. AI-enabled dialysis management systems optimize ultrafiltration, anemia control and vascular access surveillance, while generative AI and large language models offer novel capabilities for clinical documentation, triage and patient education. In transplantation, AI applications span organ allocation, dynamic graft monitoring and digital pathology-assisted rejection classification, with validated tools such as the iBox system gaining regulatory recognition. Implementation challenges include data heterogeneity, bias, interpretability, regulatory uncertainty and workflow integration. Looking ahead, multimodal integration of imaging, pathology and multi-omics data may support biologically informed precision nephrology. Reinforcement learning, digital twins and ambient intelligence are emerging as adaptive decision-support paradigms rather than autonomous care systems. Regulatory frameworks are evolving to accommodate adaptive algorithms, underscoring the need for clinician engagement in model development, validation and deployment. As AI matures from pilot to practice, nephrologists who embrace and help shape these tools will lead the transition toward a more personalized, efficient and equitable future for kidney care.

## INTRODUCTION

Artificial intelligence (AI) has reshaped modern medicine by improving diagnostic accuracy, predictive analytics, workflow efficiency and enabling AI-driven personalized care [1, 2]. Machine learning (ML) approaches facilitate the integration of large-scale clinical, imaging, and molecular data to support earlier disease detection, individualized risk stratification and dynamic treatment optimization across medical specialties [2–4]. Beyond diagnostic applications, AI has enhanced operational efficiency through automated documentation, clinical decision support (CDS) and population-level surveillance, while advancing personalized medicine frameworks that tailor interventions to patient-specific biological and clinical profiles [4–6]. These developments provide important context for the expanding role of AI in nephrology, a field characterized by complex physiology, heterogeneous disease trajectories and data-intensive decision-making.

Nephrology encompasses a uniquely complex domain at the intersection of integrative physiology and heterogeneous disease processes [4, 7]. More than 850 million people worldwide are affected by kidney disease, with chronic kidney disease (CKD) now ranking as the 12th leading cause of death. Despite its global burden, kidney disease often remains underdiagnosed or is detected late, owing to its insidious onset, subtle clinical manifestations, and multifactorial pathophysiology. These challenges render nephrology a compelling arena for innovation driven by AI [1].

AI techniques, including ML and large language models (LLMs), enable the integration of high-dimensional clinical and biological data to support early detection, risk stratification and personalized therapy. In nephrology, these tools address persistent limitations such as delayed recognition of acute kidney injury (AKI), suboptimal CKD progression modeling, and inefficiencies in dialysis and transplant workflows [4]. Yet clinical implementation has lagged, constrained by limited generalizability, lack of interpretability, regulatory uncertainty and poor alignment with existing clinical systems.

This review critically examines the state of AI in nephrology across AKI, CKD, dialysis, transplantation and renal imaging. While prior reviews have largely emphasized algorithm development and predictive performance [1, 4, 8–11], this work adopts a complementary, implementation- and systems-oriented perspective, focusing on how AI tools are integrated, governed and sustained in routine clinical practice. We emphasize validation rigor, workflow integration, regulatory and reimbursement considerations, economic feasibility and equity as key determinants of real-world clinical impact, with the goal of guiding clinicians, researchers and policymakers in advancing AI-enabled tools from experimental development to everyday kidney care.

**Figure 1:**
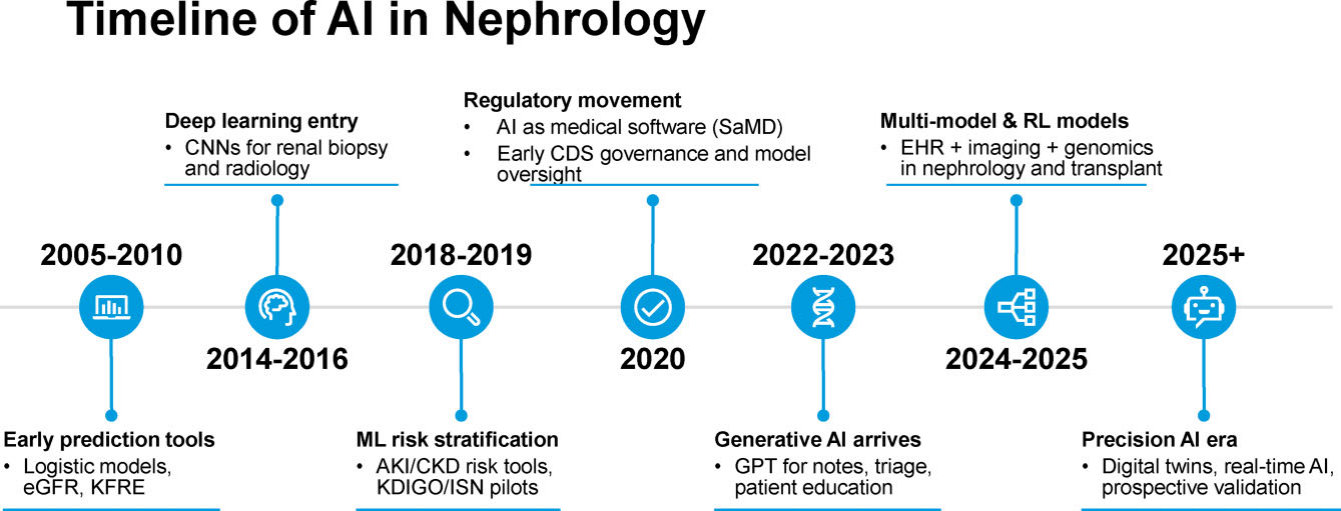
Timeline of AI in nephrology. The figure outlines key developments in nephrology AI, from early rule-based prediction tools to deep learning for imaging, ML-based risk models, regulatory approvals and the clinical adoption of generative AI. Recent advances include multimodal and reinforcement learning models, with the field now entering a precision AI era focused on digital twins, real-time validation and implementation. GPT, Generative Pretrained Transformer; ISN, International Society of Nephrology; RL, reinforcement learning.

The development of AI in nephrology has accelerated markedly over the past two decades (Fig. 1), progressing from rule-based tools such as estimated glomerular filtration rate (eGFR) calculators and the Kidney Failure Risk Equation (KFRE) to deep learning models for histopathology and radiologic interpretation. The emergence of predictive models for AKI and CKD around 2018–19 and the regulatory approval of image- and language-based tools in the early 2020s mark key inflection points. More recently, generative AI and digital twin systems have enabled real-time clinical applications in documentation, patient education and transplant monitoring. This evolution offers critical context for understanding capabilities and anticipating future directions in precision nephrology.

Although many AI models in nephrology demonstrate strong retrospective predictive performance, high accuracy alone does not ensure meaningful clinical impact [1]. Across AKI, CKD, dialysis, transplantation and renal imaging, a recurring challenge is the disconnect between algorithmic performance in retrospective or controlled settings and implementation in real-world settings with improvement in outcomes [1]. Risk stratification models may accurately predict disease progression or complications yet fail to influence care when outputs are not actionable or aligned with clinical workflows. Similarly, AI tools in dialysis and transplantation may achieve robust discrimination while offering limited incremental value in the absence of integration into longitudinal care pathways or prospective evaluation.

These limitations underscore that clinical impact depends less on marginal gains in accuracy than on validation across diverse populations, interpretability, workflow integration and demonstration of benefit in pragmatic studies that assess clinical adoption, implementation outcomes, behavior change and patient-centered outcomes. Without careful and responsible implementation testing, AI systems risk contributing to alert fatigue, inequitable care or resource misallocation, highlighting the need for balanced assessment that considers null findings and context-specific limitations alongside technical performance.

## AI APPLICATIONS IN AKI

AKI affects 10%–15% of hospitalized patients and over 50% of critically ill patients, driving excess risk of CKD, dialysis dependence and mortality [12, 13]. Traditional diagnosis relies on serum creatinine and/or urine output, which are delayed and indirect markers of kidney function that limit opportunities for early intervention. AI offers the potential to predict, detect and stratify AKI earlier in its course, enabling timely and targeted care, particularly in high-risk settings such as the intensive care unit (ICU), perioperative care and complex comorbid populations [6]. Predictive models for moderate to severe AKI [Kidney Disease: Improving Global Outcomes (KDIGO) stage 2 or 3] have achieved strong performance in multinational datasets, but generalizability remains a challenge, especially outside high-acuity settings [5, 7, 10, 11, 14–26] (Table 1).

**Table 1:** Clinical utility and limitations of AI applications in AKI.

AI application area	Example models/studies	Key benefits	Limitations/challenges
Early prediction [5, 10, 14, 15]	Epic risk model; multimodal DL	Forecast AKI 12–48 h before onset; supports preventive care	Low PPV, poor calibration, alarm fatigue
Real-time ICU decision support [7, 11]	Multimodal dashboards integrating labs, vitals, CRRT data	Continuous monitoring guides fluid and dose adjustments	Requires seamless EHR integration; clinician trust
Subphenotyping [16–21]	Unsupervised clustering (transient vs sustained injury)	Enables personalized therapy; identifies prognostic groups	Limited prospective validation; risk of overfitting
Drug dosing optimization [22–24]	Vancomycin XGBoost models	Predicts supra-/subtherapeutic levels; reduces toxicity	Not widely implemented; integration barriers
Prediction of post-AKI complications [25]	MAKE predictive model	Enables post-AKI and post-discharge risk classification	No prospective or implementation evaluation
Post-AKI remote monitoring [26]	Mayo Clinic RPM pilot	Feasible follow-up; potential reduction in emergency visits	No significant effect on readmissions; unclear cost-effectiveness

DL, deep learning; MAKE, major adverse kidney events (composite of death, dialysis dependence and drop in baseline eGFR ≥50%); PPV, positive predictive value; RPM, remote patient monitoring.

**Figure 2:**
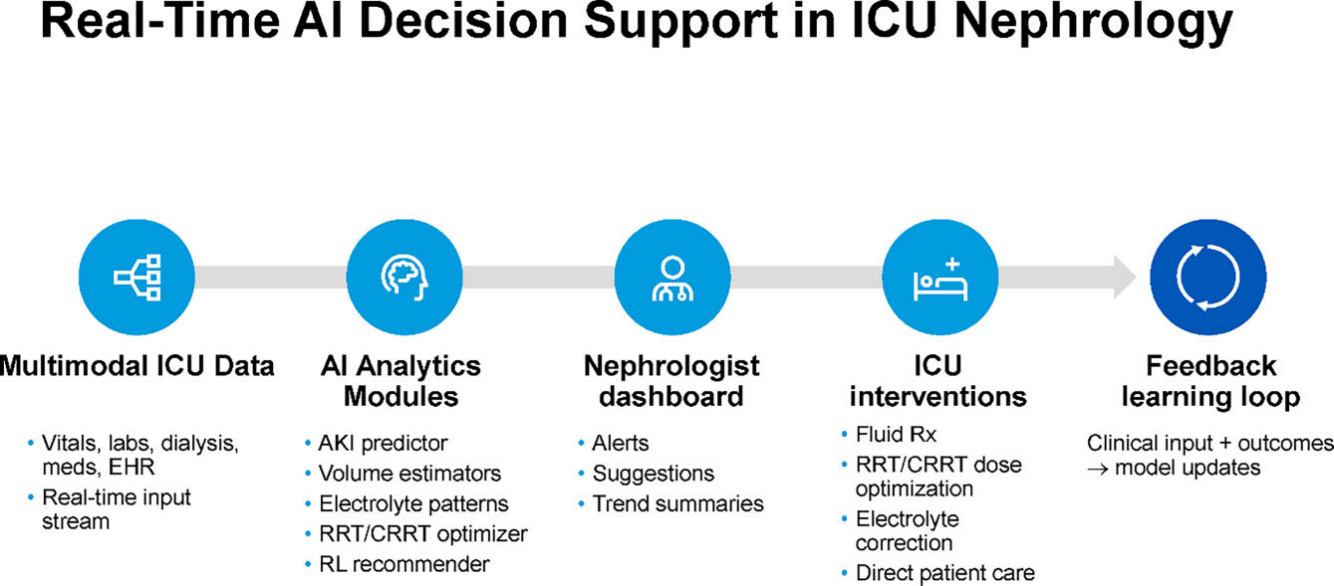
Real-time AI decision support in ICU nephrology. AI systems process continuous input from multimodal ICU data, including vitals, laboratory values, EHR information and RRT data to generate predictive and prescriptive analytics. These outputs are displayed through nephrologist dashboards and linked to ICU interventions, such as fluid management or RRT dose adjustment. A feedback loop that incorporates clinician actions and patient outcomes informs ongoing model refinement and personalization. RL, reinforcement learning; Rx, prescription.

ML models that use structured electronic health record (EHR) data, including laboratory results, vital signs and medication records, and, in advanced architectures, unstructured clinical notes, can forecast hospitalized AKI hours to days before onset [11, 13, 15, 27–30]. For example, the Epic Risk of Hospital-Acquired AKI model achieved an area under the receiver operating characteristic (AUROC) of 0.77 overall with a median lead time of 21.6 h, though low positive predictive value and calibration issues limit its impact. Deep learning approaches, such as the Koyner *et al*. [5] model integrating over 420 000 hospitalizations, have reported AUROCs up to 0.93 for kidney replacement therapy prediction, yet precision–recall tradeoffs remain significant in low-prevalence settings. Embedding these models into real-time clinical decision-support platforms, which process continuous ICU data streams and surface actionable insights to nephrologist’s dashboards (Fig. 2), can enable continuous risk monitoring, personalized fluid management, and indication-guided renal replacement therapy (RRT) [8, 10, 11, 14, 30–32].

Beyond early detection, AI supports subphenotyping through unsupervised clustering [16, 17], identifying biologically distinct AKI subtypes with differing prognoses and treatment responsiveness, such as transient hemodynamic versus sustained tubular injury patterns [18–21]. These insights could underpin precision therapeutics if validated prospectively [18–21]. AI is also being applied to drug dosing optimization and nephrotoxicity prevention, particularly from vancomycin, where extreme gradient boosting models have demonstrated strong discrimination for subtherapeutic and supratherapeutic levels in critically ill patients [18–21]. However, integration into routine clinical workflows and prospective implementation studies remain limited.

Real-world translation remains limited. A meta-analysis of 302 AKI prediction models (*n* = 3.8 million admissions) reported pooled AUROCs of 0.78–0.82, but 86% were at high risk of bias [10]. The ELAIA-2 randomized trial showed that AI-driven AKI alerts increased discontinuation of nephrotoxic drugs but did not improve AKI progression, RRT requirement or mortality [32]. Post-AKI remote patient monitoring programs incorporating daily vital signs and weekly laboratory assessments have demonstrated feasibility but no significant reduction in readmissions [33]. These findings illustrate how translational limitations are particularly evident in AKI care, where clinical heterogeneity and variable care pathways necessitate context-specific validation, calibration, workflow integration and economic evaluation.

## AI IN CKD

### AI-enabled risk prediction and management in CKD

**Figure 3:**
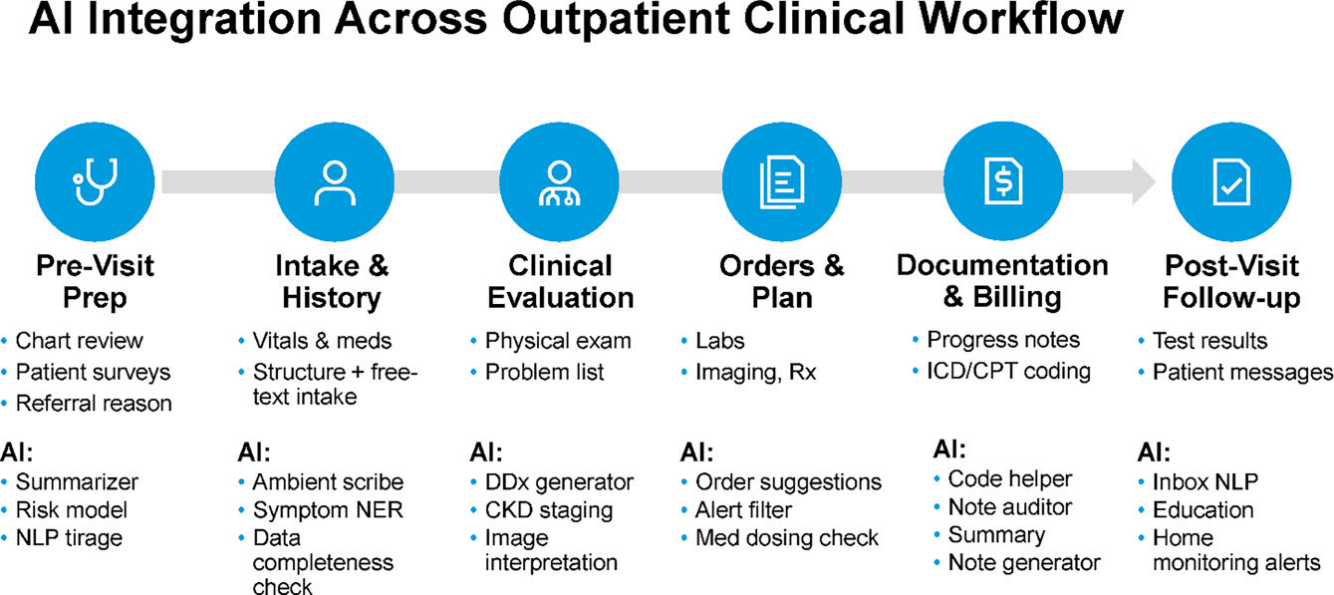
AI integration across the outpatient nephrology workflow. AI tools support end-to-end outpatient CKD care, ranging from pre-visit chart review and risk stratification to treatment planning, documentation and post-visit follow-up, thereby enhancing the timeliness, precision and scalability of decision-making. NLP, natural language processing; NER, named entity recognition; DDx, differential diagnosis; ICD, International Classification of Diseases; CPT, Current Procedural Terminology.

AI tools are increasingly integrated into outpatient nephrology workflows to support longitudinal management of CKD (Fig. 3) [3, 27]. From pre-visit chart review to post-encounter monitoring, these systems provide structured, data-driven decision support across the continuum of care.

ML-based models can identify high-risk individuals using routinely collected clinical data, reducing reliance on invasive or high-cost biomarkers [27]. For instance, Klinrisk, an externally validated prediction model, uses standard laboratory parameters to generate individualized risk trajectories in patients with type 2 diabetes [3]. It achieved an area under the curve of 0.86 for 4-year kidney failure prediction, surpassing the KFRE and KDIGO risk categories [3]. This performance illustrates the ability of AI systems to extract prognostic signals from common clinical inputs, potentially enabling scalable population-level surveillance and early intervention.

### Comparative performance and model scope

Traditional models such as KFRE demonstrate limited accuracy in early-stage CKD and often lack adaptability across diverse patient populations [34]. In contrast, modern ML approaches, particularly ensemble and deep learning algorithms, offer superior discrimination by capturing nonlinear associations and incorporating heterogeneous data types [27]. Emerging AI models span the full spectrum of CKD stages and extend applicability to at-risk populations, broadening opportunities for early and targeted interventions. Dynamic recalibration further enhances longitudinal prediction and clinical relevance.

### Clinical integration and interpretability

Real-world utility of AI in CKD care depends on seamless workflow integration and clinical interpretability. Tools such as SONIC and Trajvis provide real-time, patient-specific risk assessments embedded within the EHR [35, 36], facilitating point-of-care decision-making. Notably, several high-performing models rely on basic laboratory data, demonstrating that model simplicity can coexist with accuracy and transparency, critical attributes for clinician trust and adoption. In nephrology, simplicity coupled with interpretability may improve uptake without sacrificing clinical effect.

## AI-ENHANCED DIALYSIS MANAGEMENT

### ML-guided anemia control

**Figure 4:**
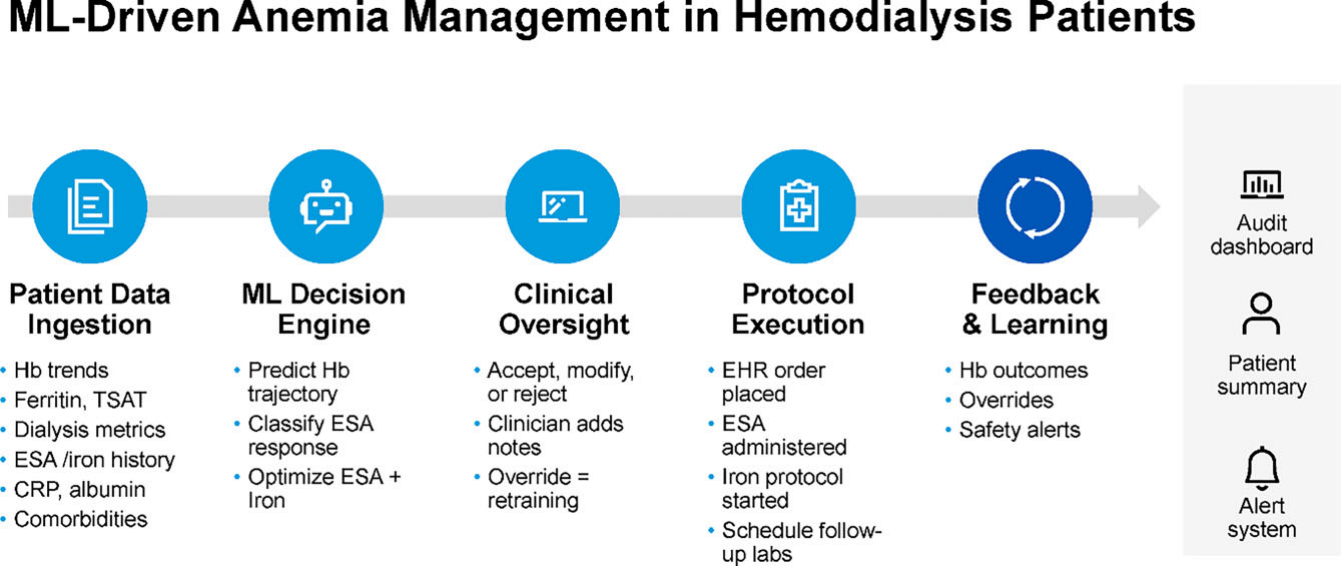
ML-driven anemia management in chronic hemodialysis. A closed-loop system processes structured patient data, including hemoglobin levels, iron indices and comorbidities to generate personalized ESA and iron dosing recommendations. Clinician review and EHR-executed protocols enable real-time implementation. Outcome feedback informs model recalibration, while optional audit and alert systems support quality assurance. TSAT, transferrin saturation; CRP, C-reactive protein; Hb, hemoglobin.

The Anemia Control Model (ACM), implemented in over 100 dialysis centers since 2013 [37], supports erythropoiesis-stimulating agent (ESA) and iron dosing through individualized, data-driven algorithms [38]. In observational analyses, ACM-guided care has been linked to a 25% reduction in ESA use, a 12% decrease in hospitalization rates and no reported safety concerns over a 10-year period [37, 39]. These findings suggest that ML can standardize complex treatment protocols and improve clinical and economic outcomes in chronic dialysis care [40]. To facilitate ML-driven anemia titration, several systems incorporate real-time hemoglobin trajectories, iron parameters, ESA responsiveness and comorbidity profiles [37, 40]. Most are deployed within a closed-loop framework that integrates clinician oversight, EHR-based protocol execution and feedback-driven model refinement. Figure 4 outlines a modular architecture for anemia management in hemodialysis, including structured data input, predictive modeling, human-in-the-loop validation and EHR-based order generation. Auxiliary modules, such as alerting and audit dashboards, enhance transparency, traceability and safety monitoring.

### Real-time prediction of intradialytic hypotension

Intradialytic hypotension (IDH) is a prevalent and clinically significant complication of hemodialysis. Recent models using recurrent neural networks, gradient-boosted trees (e.g. Extreme Gradient Boosting, XGBoost) and temporal fusion transformers have demonstrated the ability to anticipate IDH events up to 60 min in advance, with AUROC values often exceeding 0.85 across reported cohorts and prediction horizons [41–44]. Key predictors include pre-dialysis systolic blood pressure, ultrafiltration rate, interdialytic weight gain, previous IDH episodes and real-time hemodynamic trends [41–44]. Several centers have piloted AI-based CDS dashboards that deliver individualized risk alerts and recommend preventive measures, contributing to reduced IDH incidence and improved hemodynamic stability [41, 42].

### Optimizing chronic modality selection and home dialysis uptake

A national analysis of nearly 200 000 de-identified dialysis patients using over 750 clinical, demographic and laboratory variables yielded a predictive model to inform chronic dialysis modality selection [45, 46]. The model achieved AUROC values of 0.80 for home versus in-center dialysis selection, and 0.75–0.79 for distinguishing among peritoneal dialysis, home hemodialysis and conventional in-center modalities [45, 46].

When regional best practices were extrapolated through simulation modeling, projected national home dialysis utilization increased from 17.3% to approximately 28% [46]. These findings support the potential of AI-guided decision support tools to align modality selection with patient preferences and expand access to home-based therapies [46].

## AI IN KIDNEY TRANSPLANTATION

AI is transforming kidney transplantation by enhancing donor–recipient matching, post-transplant monitoring, diagnostic interpretation, immunosuppressive therapy and long-term care infrastructure [47]. From advanced ML algorithms outperforming traditional allocation models to digital pathology and AI-guided medication dosing tools, these innovations support a shift toward precision medicine.

### AI-optimized organ allocation

AI-driven tools are reshaping donor-recipient matching [48], offering improvements over conventional models such as the Model for End-Stage Liver Disease (MELD) score, which is primarily used in liver transplantation [49]. ML algorithms, including XGBoost and the Optimal Prediction of Mortality (OPOM) model, integrate a broader set of variables, including comorbidities, immunologic markers [e.g. human leukocyte antigen (HLA) compatibility, panel-reactive antibody levels] and demographic characteristics [50]. These models have demonstrated superior accuracy in predicting post-transplant survival, supporting more effective organ allocation and lower discard rates.

By enabling real-time, multivariable optimization, AI-based systems in selected contexts and for specific endpoints outperform traditional rule-based algorithms with respect to predictive utility and efficiency, and offer adaptability to dynamic organ supply, while requiring explicit consideration of tradeoffs between utility, equity and transparency in allocation decisions.

### AI in post-transplant monitoring

Long-term graft surveillance is essential to optimizing kidney transplant outcomes. The iBox system [51], a validated AI-based prognostic model, predicts allograft survival up to 10 years after transplantation and is the first tool to receive qualification as a surrogate endpoint from the European Medicines Agency and under specific US Food and Drug Administration (FDA) regulatory pathways. iBox integrates longitudinal immunologic, clinical and functional parameters to estimate individualized survival probabilities and inform treatment decisions [52]. Complementing this approach, the Dynamic Integrative System for Predicting Outcome (DISPO) enables real-time recalibration of patient-specific risk by incorporating intra-patient variability and clinical trajectories [53]. These tools exemplify the shift toward dynamic, patient-centered surveillance in transplantation. Both models are undergoing adaptation to incorporate social determinants of health, including race, ethnicity and socioeconomic status, with the goal of supporting more equitable post-transplant care [51, 53].

**Figure 5:**
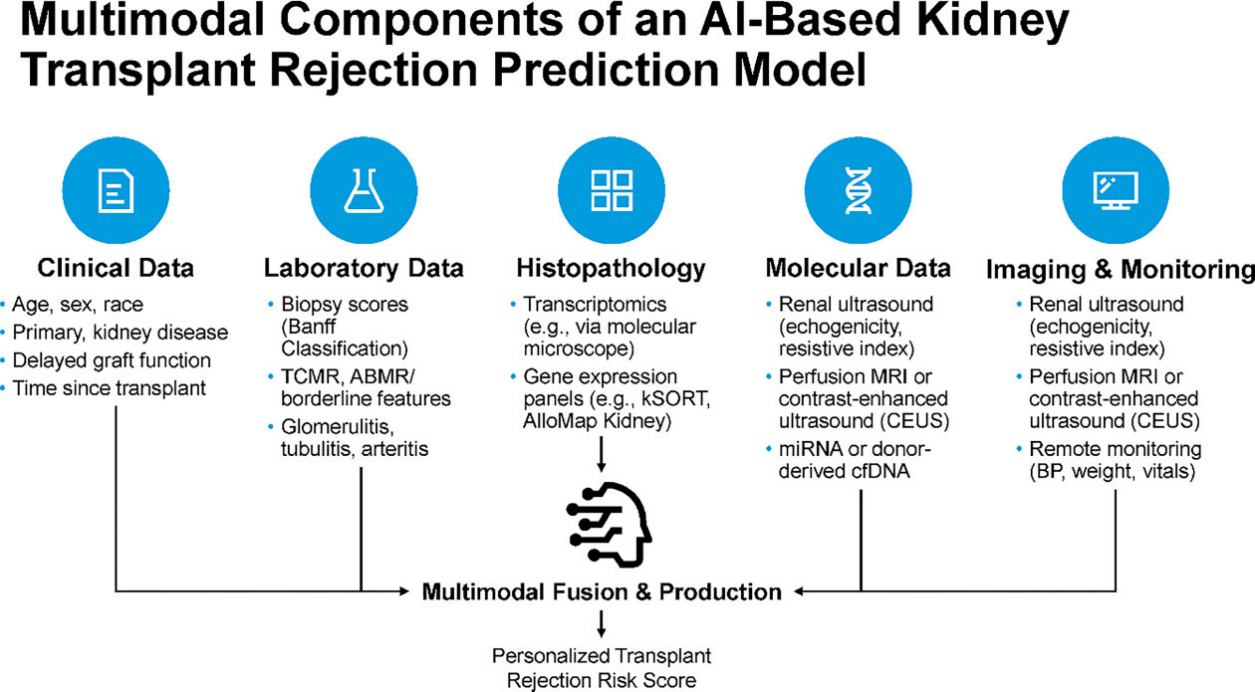
Multimodal components of an AI-based kidney transplant rejection prediction model. Data inputs include clinical characteristics, laboratory and histopathology results, transcriptomic and molecular biomarkers, imaging modalities and continuous physiologic monitoring. Multimodal fusion enables real-time, individualized rejection risk scoring. ABMR, antibody-mediated rejection; BP, blood pressure; CEUS, contrast-enhanced ultrasound; cfDNA, cell-free DNA; kSORT, Kidney Solid Organ Response Test; miRNA, microRNA; MRI, magnetic resonance imaging; TCMR, T cell–mediated rejection.

Beyond iBox and DISPO [51, 53], next-generation AI platforms increasingly rely on multimodal data fusion to improve predictive accuracy and clinical relevance (Fig. 5). These systems integrate diverse inputs, including structured clinical and laboratory data, histopathologic findings, molecular signatures, imaging studies and continuous physiologic monitoring, into unified models for individualized risk assessment. Each data modality contributes distinct insights; for example, transcriptomic and gene expression profiles reveal immune activation patterns, while imaging and wearable devices offer temporal granularity. Deep learning and ensemble methods enable these heterogeneous signals to be synthesized into real-time rejection risk scores, potentially facilitating earlier and more targeted interventions.

### Digital pathology and AI-enhanced diagnostics

AI-based digital pathology has ushered in a new era of transplant diagnostics [54, 55]. The Banff Automation System (BAS) employs convolutional neural networks (CNNs) to analyze biopsy slides and automatically classify histologic lesions, including glomerulitis, peritubular capillaritis and interstitial inflammation [56]. In a multicenter study, BAS reclassified approximately 30% of antibody-mediated rejection and 54% of T cell–mediated rejection cases, underscoring its clinical relevance [56]. Ensemble learning models and vision transformers further enhance diagnostic precision by integrating histologic, transcriptomic and clinical data [57]. Spatial transcriptomics adds an additional layer of granularity, enabling mapping of gene expression patterns within specific tissue microenvironments [58]. Applied to kidney transplant biopsies, this technology has uncovered macrophage-driven inflammation in borderline rejection and challenged T cell–centric paradigms [59, 60]. Emerging studies also highlight the potential role of AI-powered pathology in organ procurement, particularly for rapid viability assessment and donor kidney quality evaluation, which may streamline allocation and improve utilization [47, 54, 55].

### AI-driven personalization of immunosuppression

Immunosuppressive therapy remains the cornerstone of graft preservation, yet between-patient variability complicates management [61]. AI-based tacrolimus dosing models integrate pharmacogenomic, clinical and demographic data to improve drug bioavailability predictions and reduce intrapatient variability, thereby lowering the risk of under- or over-immunosuppression [62].

*In silico* drug repurposing methods harness AI to identify existing immunomodulatory agents with potential utility in transplantation [47]. For example, ibrutinib, originally approved for B-cell malignancies, has demonstrated promise in modulating T-cell responses and cytokine profiles relevant to rejection. Future applications include digital twins and LLMs, which may simulate individual immune responses and optimize immunosuppressive regimens *in silico*. These virtual platforms can potentially test drug combinations and tapering strategies before real-world implementation [47].

### AI-augmented transplant care

AI technologies are reshaping not only clinical decision-making but also the operational infrastructure of transplant care. Integrated platforms, often referred to as AI-augmented transplant clinics, aim to consolidate predictive models, automation tools and real-time monitoring within a unified clinical ecosystem [63, 64]. Digital twin frameworks exemplify this approach by generating dynamic, computational simulations of individual patients to inform pre-operative planning and post-transplant management. These systems ingest multimodal data, including laboratory results, genomic information, imaging, wearable sensor outputs and psychosocial metrics, to construct real-time models that simulate physiologic responses and test therapeutic strategies. At present, however, most nephrology digital twin applications remain at a pre-clinical or proof-of-concept stage, with limited prospective validation or real-world deployment. By enabling prospective evaluation of immunosuppression regimens, surgical risks and graft function trajectories, digital twins may support more individualized and anticipatory care.

**Figure 6:**
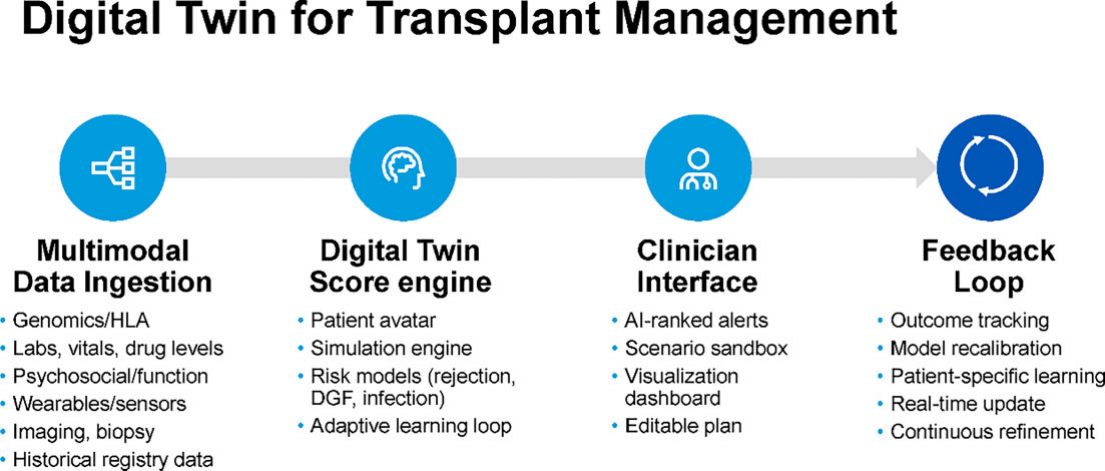
Digital twin framework for transplant management. The architecture comprises five components: data ingestion from clinical and sensor inputs; a simulation engine integrating physiologic and risk models; a clinician interface with alerts and planning tools; an outcome-based feedback loop; and a longitudinal model refinement module. The system supports individualized immunosuppression and early detection of graft dysfunction through predictive simulation. DGF, delayed graft function.

As illustrated in Fig. 6, the digital twin pipeline comprises real-time data input, a core simulation engine with embedded risk models (e.g. for rejection or delayed graft function), a clinician-facing interface for scenario exploration and alert prioritization, and an outcome-driven feedback loop for continuous model refinement. Such systems hold the potential to individualize immunosuppression, anticipate complications and support shared decision-making by simulating treatment outcomes before actual implementation.

Real-time alerts from wearable biosensors and connected devices can detect early signs of graft dysfunction or infection [47], triggering automated team-based care coordination. Although these systems remain conceptual, they represent a future in which AI augments all facets of transplantation, from the operating room to outpatient monitoring [47].

## LLM AND GENERATIVE AI IN NEPHROLOGY

LLMs and generative AI are rapidly reshaping knowledge access, CDS and communication within nephrology [7, 65–67]. Trained on extensive corpora, including biomedical literature, clinical guidelines and general-domain texts, these models enable a wide range of capabilities such as real-time summarization of complex clinical narratives, generation of patient education materials, and support for dialysis management and documentation tasks.

**Figure 7:**
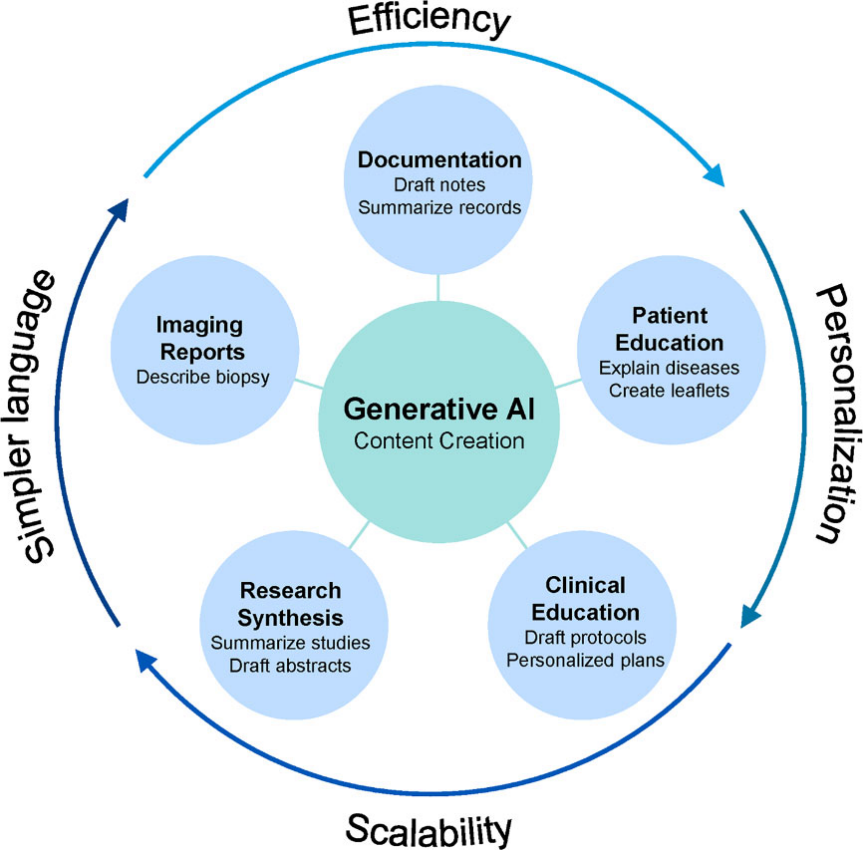
Generative AI applications in nephrology. This diagram summarizes key use cases of generative AI in nephrology, centered on content creation. Applications include documentation, imaging reports, patient and clinical education, and research synthesis—aligned with goals of efficiency, personalization, scalability and simplification of medical language.

Applications span multiple domains: triaging inbox messages (e.g. prioritizing urgent patient concerns), interpreting laboratory values (such as abnormal electrolytes or kidney function tests), generating imaging reports, drafting notes and supporting personalized healthcare professional and patient education (e.g. medication adherence, diet, dialysis care). As illustrated in Fig. 7, generative AI tools are being deployed to enhance efficiency, scalability and personalization across nephrology workflows. These include documentation (e.g. record summarization), biopsy interpretation in accessible language, research protocol generation and tailored disease education. These functions align with core value domains by simplifying complex language, enabling scalable content generation and personalizing outputs into clinical context.

**Figure 8:**
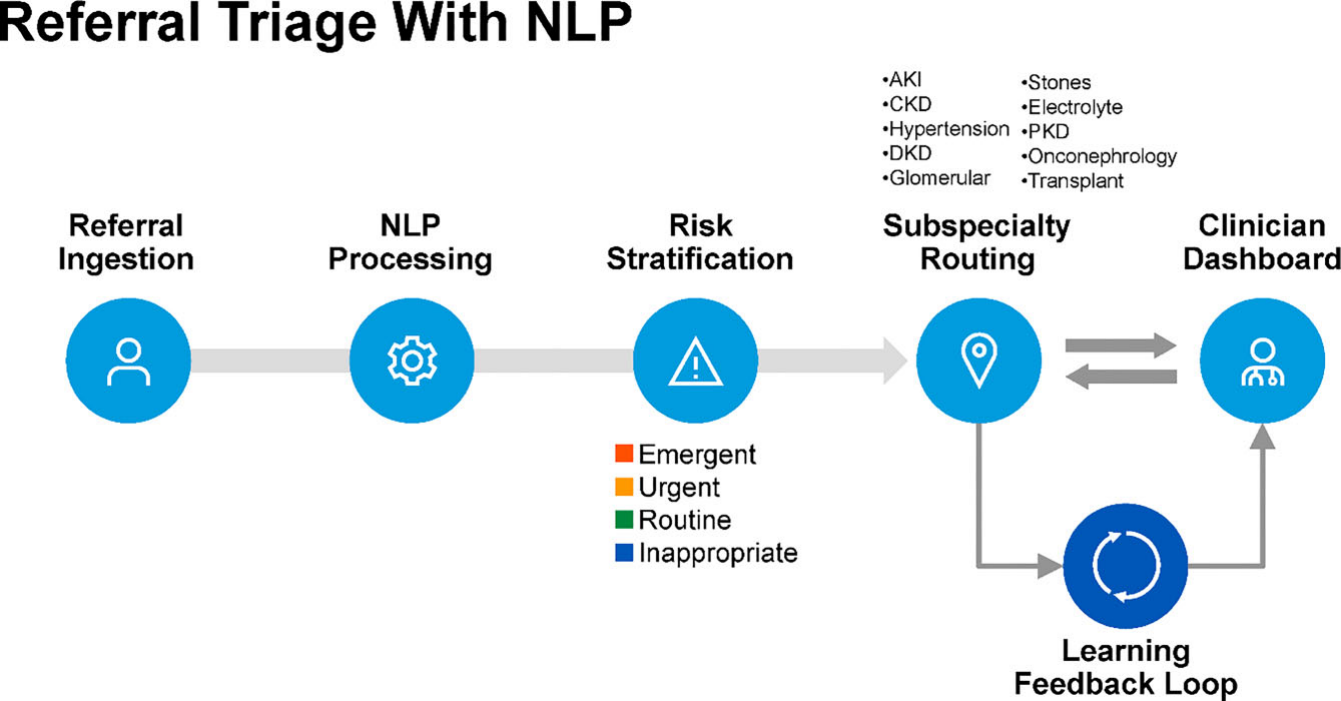
NLP-driven referral triage workflow in nephrology. NLP algorithms automatically extract clinical concepts from free-text referrals, enabling real-time risk stratification (e.g. emergent, urgent, routine or inappropriate) and subspecialty routing. A clinician-facing dashboard supports final triage decisions and oversight. A continuous feedback loop facilitates model refinement, incorporating triage outcomes, clinician overrides, and learning signals to improve system performance over time. DKD, diabetic kidney disease; NLP, natural language processing; PKD, polycystic kidney disease.

Ambient documentation represents a notable frontier in this domain. Generative AI tools such as Abridge have been piloted within EHRs (e.g. Epic-based) to improve inpatient workflows across physician, nursing and allied health teams, and convert clinician–patient dialogue into structured notes in real time. In a recent pragmatic randomized clinical trial [68], ambient AI scribes were associated with reductions in documentation burden and modest improvements in burnout-related measures, including task load and work exhaustion, although effects varied by platform and were observed primarily in secondary endpoints. Nonetheless, human oversight remains essential to mitigate model hallucinations and ensure documentation fidelity. One specific application is referral triage, where natural language processing–based systems extract and classify free-text referral data to assign urgency and route cases to appropriate subspecialists, improving prioritization, access and equity (Fig. 8). Generative AI models such as GPT-4 and Bard are increasingly integrated into these workflows, often augmented with retrieval-augmented generation (RAG) systems to enhance factual grounding.

Recent reports in nephrology have identified multiple use cases for LLMs, spanning workflow optimization, lab interpretation, renal dietary guidance and patient education [7, 69]. GPT-4 achieved 90%–94% accuracy in managing continuous renal replacement therapy (CRRT) alarms in simulated and benchmarked task settings, and significantly improved readability of educational materials in experimental evaluations [7, 69]. RAG and chain-of-thought approaches further enhanced diagnostic accuracy in complex conditions, such as diabetes insipidus. Bard AI and GPT-4 performed best in nutrient classification, while Microsoft Copilot led in lab result interpretation. However, all models remained sensitive to prompt phrasing and lacked real-world validation [69].

Despite encouraging performance, notable limitations include minimal external validation, variable methodological rigor and lack of fairness assessment across demographic groups. Many models struggled with complex, guideline-driven queries and were prone to oversimplification. Broader issues—including data privacy, premium vs free access disparities and unclear implementation frameworks—remain unresolved. Crucially, few studies assessed clinical outcomes. Thus, while generative AI shows promise for augmenting education and administrative workflows, clinical integration remains premature pending prospective validation and implementation and effectiveness testing.

## AI IN RENAL IMAGING AND PATHOLOGY

AI is offering advances in both imaging acquisition and interpretation, as well as in histopathologic assessment [70, 71]. Its applications span technical optimization, diagnostic precision and workflow efficiency, with the potential to reshape how renal diseases are diagnosed and managed.

### AI in image acquisition

AI-driven algorithms now facilitate several aspects of image acquisition. In modalities such as magnetic resonance imaging (MRI), ML systems can automatically optimize signal-to-noise ratios, adjust positioning and reduce scan duration [72]. These capabilities reduce operator dependency and inter-reader variability while improving diagnostic yield [72]. Point-of-care ultrasound systems increasingly incorporate real-time AI guidance to standardize imaging planes and reduce scan times [73, 74]. Such tools may extend high-quality imaging to under-resourced settings where operator expertise is limited [72, 74].

## AI IN TISSUE SAMPLING AND ADEQUACY ASSESSMENT

Histopathologic evaluation remains central to nephrology diagnostics, yet renal biopsies are frequently limited by inadequacy rates as high as 22% [75]. To mitigate this, AI-based platforms are being developed to support real-time biopsy adequacy assessment [54]. Early-stage AI tools have been developed to analyze smartphone-captured images, enabling identification of glomeruli and differentiation between cortical and medullary regions—features critical to assessing renal biopsy adequacy [76–79]. These platforms offer significant potential for bedside deployment, particularly in settings lacking immediate pathology support. By improving adequacy assessment, these applications may reduce the need for repeat biopsies, lower procedural costs and accelerate time to diagnosis.

## AI IN IMAGE INTERPRETATION AND COMPUTATIONAL PATHOLOGY

Beyond acquisition, AI enhances image interpretation. CNNs and U-Net architectures have achieved intersection-over-union scores exceeding 0.89 for renal structure segmentation [80–82]. These capabilities enable automated measurement of cortical thickness, renal volumes and other morphometric indices, supporting quantitative and prognostic imaging in nephrology [83].

In renal pathology, computational approaches are increasingly applied to morphologic feature detection, disease staging and prognostic modeling [84, 85]. When integrated with digital whole-slide imaging, deep learning algorithms can automate the recognition of glomerulosclerosis, immune complex deposition, tubular atrophy and interstitial fibrosis [85]. Advanced image analysis can quantify features such as peritubular capillary density, offering prognostic information that extends beyond conventional histopathologic reporting [86]. Clinical deployment, however, remains constrained by the limited availability of large, well-annotated datasets for rare diseases, variability in tissue processing, and the absence of standardized, multicenter image repositories. Broader adoption will require structured training in computational pathology and the development of clear regulatory frameworks [85].

**Figure 9:**
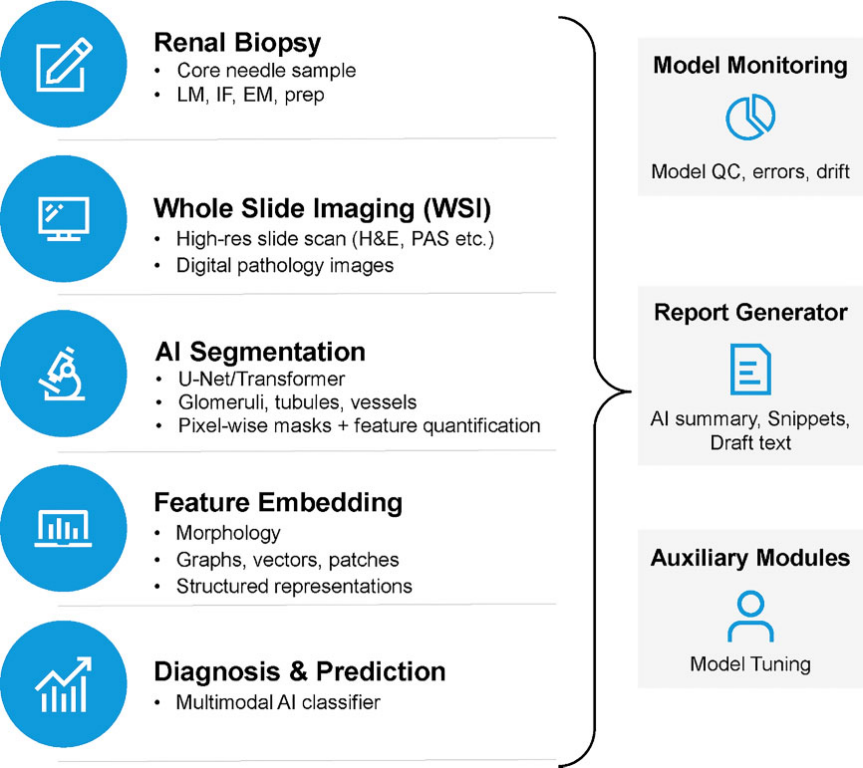
Biopsy-to-AI diagnostic pipeline. This schematic outlines the multimodal workflow for AI-assisted renal pathology. Input sources include histologic, immunofluorescence and electron microscopy images, along with clinical metadata. Whole-slide imaging enables AI-based segmentation and morphological feature extraction. These feed into multimodal classification models for diagnostic labeling, prognostic inference, and pathology report drafting. A feedback loop allows pathologist input to refine and retrain the model, supporting continuous learning and performance optimization. EM, electron microscopy; H&E, hematoxylin and eosin; IF, immunofluorescence; LM, light microscopy; PAS, periodic acid–Schiff; QC, quality control; WSI, whole-slide imaging.

Emerging AI frameworks leverage multimodal data (Fig. 9), including light microscopy, immunofluorescence, electron microscopy and structured clinical inputs, to enable end-to-end diagnostic modeling. These systems perform segmentation, extract morphologic features and generate diagnostic or prognostic labels, with feedback loops that support continuous model refinement. In addition, non-invasive diagnostic AI tools are also emerging. Deep learning models such as DeepDKD [87], trained on retinal images, have achieved AUROC values of 0.842 for detecting diabetic kidney disease and 0.906 for differentiating diabetic nephropathy from other glomerular diseases [87]. These models exemplify how AI may support early risk stratification without requiring renal biopsy.

Despite promising advances, broad clinical integration of AI in nephrology faces persistent challenges. These include the need for diverse, annotated datasets; regulatory uncertainty; and interpretability of algorithm outputs. Efforts to establish quality-control pipelines, standardize image preprocessing and train nephrologists in AI fundamentals will be pivotal. As these barriers are addressed, AI-based tools are poised to reduce diagnostic variability, facilitate earlier disease detection and enable precision nephrology.

## WEARABLE AND IMPLANTABLE DEVICES IN KIDNEY CARE

Recent advances in wearable and implantable bioelectronic systems are transforming kidney care by enabling non-invasive, continuous monitoring of physiological and biochemical parameters [88]. These technologies offer the potential to detect kidney dysfunction earlier than conventional laboratory methods, reduce reliance on invasive testing, and support personalized, real-time clinical management [88].

### AI-enhanced electrocardiography for hyperkalemia surveillance

Kardio-Net is a deep learning model trained on over 290 000 electrocardiograms (ECGs), optimized to detect severe hyperkalemia (serum potassium >6.5 mmol/L) [89]. It can analyze both standard 12-lead ECGs and single-lead smartwatch tracings (e.g. Apple Watch). In end-stage kidney disease populations, the model achieved an AUROC of 0.85–0.90. Kardio-Net enables continuous potassium monitoring between dialysis sessions and can issue real-time alerts for critical electrolyte disturbances [89]. However, sensitivity is reduced in single-lead configurations, and prospective validation remains limited.

### Saliva-based biosensors

Intraoral platforms, such as dental retainers and mouthguards, are being developed to measure creatinine and uric acid in saliva [88]. These wearable devices enable passive, noninvasive biochemical monitoring and may be particularly useful for monitoring hydration status and electrolyte imbalances in CKD; they may also have a role in the care of patients in rural settings where nephrology specialists and laboratory access are limited.

### Microneedle sensors for interstitial fluid analysis

Minimally invasive microneedle arrays provide access to interstitial fluid without penetrating blood vessels or nerves [88]. These systems allow continuous electrochemical detection of key analytes, including creatinine, potassium and sodium, with high temporal resolution and minimal patient discomfort.

### Cuffless blood pressure monitoring using photoplethysmography and ECG

Wearable blood pressure (BP) monitors leveraging photoplethysmography and ECG are undergoing validation for cuffless, continuous BP assessment [90]. These devices are favored by patients due to improved comfort and convenience [90]. However, concerns remain regarding measurement accuracy across diverse skin tones, body types and clinical conditions. Institute of Electrical and Electronics Engineers (IEEE) and International Organization for Standardization (ISO) protocols have been proposed to standardize validation and performance metrics.

### Implantable technologies

#### Temperature-sensing implants for early detection of allograft rejection

In preclinical and early translational studies, temperature-sensing implants have detected kidney transplant rejection 2–3 days earlier than changes in serum creatinine or blood urea nitrogen [88]. These devices quantify tissue temperature fluctuations associated with inflammatory rejection, providing a promising adjunct to conventional post-transplant monitoring strategies [91].

#### Optical sensors for renal perfusion monitoring

Implantable optical devices capable of measuring tissue oxygen saturation (StO₂) within the renal parenchyma are under investigation for perioperative and critical care applications [88]. These sensors may prevent complications such as renal artery thrombosis or mitigate ischemic injury by detecting early perfusion deficits.

## IMPLEMENTATION CHALLENGES AND CONSIDERATIONS

### Data quality, bias, equity and generalizability

Data quality and representativeness remain foundational determinants of AI performance, generalizability and equity in nephrology [92]. As in other clinical domains, AI systems trained on biased, incomplete or context-specific datasets may exhibit differential performance across populations and care settings, with downstream implications for fairness and scalability [93]. These limitations are particularly salient in nephrology, where disease heterogeneity, fragmented longitudinal care and variability in practice environments challenge robust model development, deployment and implementation [94, 95].

### Bias, fairness and equity considerations

Differential algorithmic performance across race, ethnicity, socioeconomic status and care settings has been documented in nephrology and related clinical domains, raising concerns regarding fairness and the potential amplification of existing health disparities [1, 2, 93]. Such differences often reflect biases embedded in training data rather than biological variation. AI-based decision support may also introduce automation bias, whereby clinicians over-rely on model outputs despite contextual misalignment, particularly when models are trained on population-specific or suboptimal datasets [94, 95].

Real-world experience underscores that biased AI outputs can meaningfully influence clinical decision-making [1, 2, 93]. For example, AKI prediction models developed primarily in intensive care settings may perform inconsistently in community hospitals, while transplant risk prediction tools relying heavily on longitudinal utilization or laboratory data may disadvantage patients with fragmented care histories [5–7, 47, 51]. Comparable patterns observed in cardiovascular and critical care risk models further highlight the broader relevance of these concerns to nephrology practice [1]. These observations reinforce the need for bias-aware model development, external validation across diverse settings and transparent reporting of subgroup performance to support equitable AI deployment in kidney care.

### Generalizability across modalities and care settings

Generalizability challenges extend beyond structured clinical data to renal imaging and computational pathology, where the rarity of many kidney diseases yields only small datasets, increasing risks of overfitting and limiting external validity [96]. Variability in tissue processing, staining protocols, imaging hardware and acquisition workflows further complicates harmonization and scalability, often requiring resource-intensive curation and standardization efforts prior to multi-site deployment.

### Institutional readiness and structural constraints

Beyond data-related considerations, adoption of AI tools in nephrology is constrained by structural, institutional and workforce-related factors [1, 4]. Fragmented data infrastructure, limited access to longitudinal records and interoperability challenges across EHR vendors and dialysis platforms impede implementation and disproportionately affect community and resource-limited settings [92, 93]. Effective deployment therefore depends not only on model performance, but on institutional readiness, including governance frameworks for oversight, monitoring and recalibration, as well as clinician readiness shaped by AI literacy, trust and workflow alignment [1, 4, 6, 97, 98].

Drawing on institutional experience across large academic health systems, a pragmatic implementation roadmap for AI deployment in nephrology includes: (i) prioritizing narrowly defined, high-utility clinical use cases; (ii) early engagement of clinicians, informaticians and governance stakeholders; (iii) local robust validation prior to deployment; (iv) direct embedding of outputs within EHR workflows; and (v) post-deployment monitoring for performance drift, bias and unintended consequences [6, 24, 99–101]. These principles are broadly generalizable and emphasize that AI readiness is an institutional property rather than a model-specific attribute [1, 93, 94].

### Economic and sustainability considerations

Economic considerations further influence the equitable and sustainable translation of AI in nephrology [1, 4]. Emerging evidence suggests that AI-enabled interventions may support cost savings and resource optimization in high-burden settings such as AKI, dialysis and transplantation, although effects on hard clinical outcomes remain variable. Observational studies have linked AI-driven AKI risk stratification and medication safety tools to reduced nephrotoxin exposure, shorter ICU stays, and more efficient allocation of nephrology consultation resources. In chronic dialysis care, ML-guided anemia management and prediction of intradialytic complications have been associated with reductions in medication use, treatment interruptions and hospitalization rates.

Despite these promising signals, few AI applications have undergone formal evaluation of cost-effectiveness, reimbursement feasibility or long-term sustainability [1, 4]. Implementation costs related to data infrastructure, EHR integration, governance oversight and ongoing model monitoring may offset downstream savings, particularly outside well-resourced centers. Incorporation of health economic endpoints into future validation and implementation studies will be essential to support responsible and equitable adoption of AI-enabled kidney care.

### Open-source versus commercial AI systems

Meaningful distinctions also exist between open-source and commercial AI systems in nephrology [1, 92–94]. Open-source tools offer transparency, reproducibility and adaptability, enabling independent auditing and local customization, but typically require substantial institutional expertise and informatics support [4, 6, 97, 98]. In contrast, commercial platforms provide integrated deployment pathways, regulatory support and vendor accountability, yet may limit transparency, flexibility and independent external validation [14, 102–104]. Hybrid approaches that combine open-source models with regulated commercial infrastructures may offer a pragmatic balance between innovation, scalability and governance. Careful consideration of these tradeoffs is essential when selecting AI tools for clinical deployment [1, 98, 105, 106].

### Validation and real-world translation

Beyond data quality and bias, limited external and real-world validation remains a major challenge across AI applications in nephrology [1, 4]. Many published models rely on single-center or retrospective datasets with internal cross-validation but lack independent external testing, constraining confidence in generalizability and clinical readiness [5, 7, 10, 11, 14–26]. Models that perform well in development cohorts frequently exhibit degraded calibration or utility when applied across institutions, populations or care settings, reflecting differences in case mix, practice patterns and data infrastructure [6, 7].

Importantly, lack of external validation does not invalidate prior AI studies [5, 7, 10, 11, 14–26], but rather defines their position along the translational pathway. Internally validated models may demonstrate strong technical performance yet remain unsuitable for clinical deployment without further testing [1]. This distinction is particularly relevant in nephrology, where disease and population heterogeneity and variability in dialysis and transplant practices amplify risks of miscalibration outside highly resourced environments.

Real-world validation extends beyond dataset testing to include prospective evaluation of workflow integration, clinician interaction and downstream clinical effects [5, 7, 10, 11, 14–26]. Effective validation therefore requires assessment of how predictions are operationalized within clinical workflows, how clinicians interpret and act on model outputs, and whether deployment results in measurable improvements in patient-centered and system-level outcomes.

Advancing methodological rigor will require multi-site external validation, transparent reporting of calibration and subgroup performance, and prospective implementation studies assessing adoption, safety, equity and effectiveness [1]. As highlighted in Table 2 rigorous validation and real-world testing are foundational requirements for responsible AI deployment in nephrology rather than optional extensions [1, 3, 4, 7, 11, 13, 31, 37, 40–44, 51–54, 65–67, 84, 85, 107–152].

**Table 2:** Comparative overview of AI applications across nephrology domains.

Domain	Representative use case	Input data	Model performance (reported metrics)	Validation method	Clinical readiness
AKI [1, 4, 11, 13, 31, 107–114]	Early AKI prediction (12–48 h before onset) Predicting CRRT need or prognosticating outcomes (mortality, renal recovery) in CRRT patients	EHR laboratory results, vital signs, medications, clinical notes Retrospective EHR data: labs, vitals, demographics, clinical scores	General wards: ∼0.79–0.91 ICU: ∼0.88–0.91 CRRT need: ∼0.88–0.99 Renal recovery: ∼0.75 Mortality: ∼0.76	Retrospective with multicenter and international external validation; limited prospective validation Single-center development with limited external validation	Limited real-world adoption; alert fatigue and unclear clinical impact remain barriers Design/early validation phase; no prospective validation or real-world implementation
CKD [3, 115–124]	Predicting kidney failure or 40% eGFR decline	Demographics, laboratory results (eGFR, proteinuria/UACR, creatinine, albumin, hemoglobin), comorbidities; some models include cystatin C, electrolytes	Kidney failure (2–5 years): 0.81–0.91 40% eGFR decline: 0.67–0.68 Short-term progression (6 months): 0.85–0.93	Extensive external validation across multiple countries and healthcare systems (>1 million patients for KFRE); predominantly retrospective	Validated in research settings; limited real-world clinical implementation; barriers include workflow integration, interpretability, and regulatory approval
Dialysis [37, 40–44, 125–136]	ESA dose optimization and hemoglobin prediction IDH prediction: pre-dialysis prediction (before session) and real-time prediction (during ongoing session)	Hemoglobin, iron indices (ferritin, transferrin saturation), ESA dose, dialysis adequacy (Kt/V), demographics, comorbidities, laboratory values (albumin, CRP) Pre-dialysis models: demographics (age, sex), pre-dialysis BP, ultrafiltration target rate, interdialytic weight gain, laboratory values, prior session data (mean nadir SBP, IDH occurrence) Real-time models: time-series vital signs (BP dynamics, HR variability), elapsed dialysis time, ultrafiltration rate, machine data	Hemoglobin prediction MAE: 0.60–0.75 g/dL ESA dose recommendation accuracy: 0.72–0.87 Transfusion alert accuracy: 0.99 Pre-dialysis prediction: 0.82–0.89 Real-time prediction: 0.87–0.95 Varies by IDH definition (nadir SBP <90 mmHg vs BP decrease criteria): 0.77–0.95	International multicenter observational studies (retrospective and prospective); cohorts >110 000 patients across 12 countries Retrospective multicenter validation with large datasets (>260 000 sessions); external validation across multiple centers; prospective clinical validation extremely limited; systematic review found 87.5% of studies have high risk of bias	Deployed in selected centers; demonstrated improved outcomes and cost-effectiveness Pilot implementation at selected centers with AI dashboards; lacks widespread clinical validation and adoption; heterogeneity in models and settings limits generalizability
Transplant [51–53, 137, 138]	Long-term kidney allograft failure prediction (death-censored graft loss)	Functional parameters: eGFR, proteinuria (UPCR) Histological features: IFTA, allograft inflammation (Banff scores) Immunological markers: Anti-HLA DSA	C-index: 0.80–0.81 across international cohorts Dynamic prediction models: 0.82–0.87 (incorporating repeated measurements over time)	International multicenter external validation across 18 academic centers in Europe, USA, and South America (13 608 patients); validated in 3 randomized controlled trials and pediatric populations (20 centers)	Regulatory-qualified surrogate endpoint for clinical trials; potential for clinical decision support; limited evidence of routine clinical implementation
Kidney pathology [54, 84, 85, 139–150]	Glomerular segmentation and detection; quantification of IFTA and glomerulosclerosis; classification of glomerular pathological findings (global/segmental sclerosis, endocapillary proliferation, mesangial changes, crescents, basement membrane changes); grading of inflammation and capillaritis; disease diagnosis (IgAN, MN, FSGS, MCD, DN)	Whole-slide images from PAS, PAMS, Masson trichrome and other stains; some models incorporate clinical data, laboratory values, demographics; advanced models use multiple stains simultaneously	Glomerular detection: Dice 0.94–0.95, F-score 0.76–0.82 Global sclerosis: AUROC 0.983–0.986 Multiclass segmentation: Dice 0.80–0.84 Specific glomerular findings: AUROC 0.70–0.99 Inflammation/capillaritis grading: AUROC >0.86–0.94 Disease diagnosis (4-type GN): F1-score 0.84–0.85, precision 0.81–0.83, recall 0.88–0.89	Multicenter retrospective validation with external datasets; multicenter study with 6682 patients across 3 institutions; international validation; 99% of AI pathology studies have ≥1 area at high/unclear risk of bias; prospective validation extremely limited	Research and development phase; limited clinical deployment; barriers include workflow integration, clinical utility demonstration, regulatory approval, IT infrastructure, interpretability; only ∼9% of studies performed in clinical contexts; technology expected to enhance rather than replace nephropathologists
Generative AI [7, 65–67, 151, 152]	Ambient clinical documentation (automated note-taking); clinical note generation/summarization; discharge summaries; clinical referrals; patient messaging; medical billing and coding; diagnostic triage and differential diagnosis	Ambient audio, free-text clinical notes, EHR data, clinical scenarios/case vignettes and multimodal inputs (tabular data, text, limited imaging)	AUROC not uniformly reported; task-specific metrics vary, with documentation success reported by 53% of health systems, diagnostic accuracy up to 88% (top-3 predictions) and triage accuracy around 70% with declining performance at higher acuity	Heterogeneous benchmarking and pilot studies with fragmented designs, limited external and real-world validation, lack of standardized frameworks, and challenges due to proprietary model opacity	Ambient documentation is widely adopted with early operational success, whereas patient messaging and administrative applications show modest impact, and diagnostic and triage uses remain exploratory, requiring clinician oversight and human–AI teaming due to safety concerns

BP, blood pressure; CRP, C-reactive protein; DN, diabetic nephropathy; DSA, donor-specific antibodies; FSGS, focal segmental glomerulosclerosis; GN, glomerulonephritis; HR, heart rate; IgAN, immunoglobulin A nephropathy; IFTA, interstitial fibrosis and tubular atrophy; Kt/V, dialysis adequacy index; MAE, mean absolute error; MCD, minimal change disease; MN, membranous nephropathy; PAMS, periodic acid methenamine silver; PAS, periodic acid–Schiff; SBP, systolic blood pressure; UACR, urine albumin-to-creatinine ratio; UPCR, urine protein-to-creatinine ratio.

### Regulatory and privacy frameworks

Regulatory frameworks for medical AI underwent major updates in 2025 [102, 103]. The US FDA finalized its Predetermined Change Control Plan (PCCP) guidance, establishing the first explicit pathway for adaptive, continuously learning AI systems. Under this framework, developers must prospectively specify model components that are eligible for updates, the expected change protocols and the requirements for post-market performance monitoring. This structure enables iterative improvement while maintaining safety and regulatory compliance. In parallel, the European Union enacted the EU AI Act [153], which classifies all healthcare AI tools as high risk and requires robust transparency, risk management and human oversight. Complementary methodological frameworks, including Good Machine Learning Practice (GMLP) [154], SPIRIT-AI for trial protocols [155] and CONSORT-AI for reporting [156], support reproducibility, interpretability and international harmonization. These developments have direct relevance for high-stakes applications in critical care nephrology, including AKI prediction models, CRRT optimization algorithms and LLM-assisted reasoning systems, where adaptive behavior, auditability and real-world monitoring are essential.

**Figure 10:**
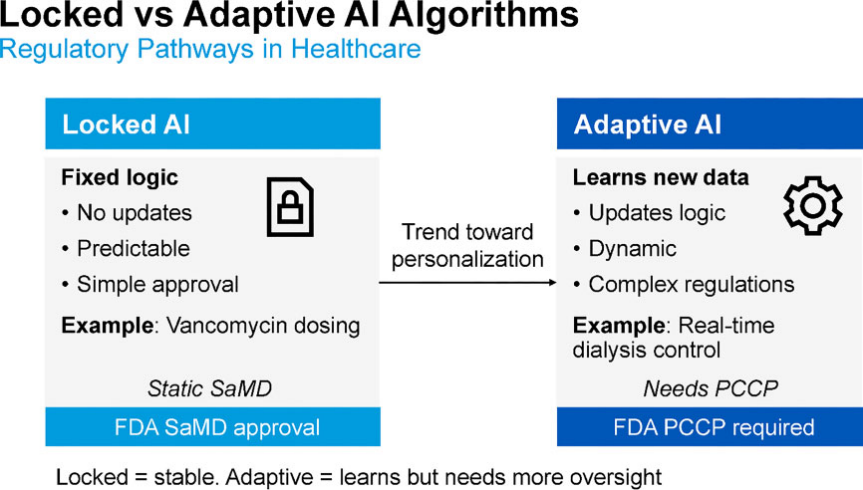
Locked vs adaptive AI algorithms: regulatory pathways in healthcare. Locked AI systems use fixed logic, making them predictable and eligible for streamlined approval (e.g. static vancomycin dosing tools). Adaptive AI systems continuously learn and update their logic, enabling greater personalization but requiring complex regulatory oversight under mechanisms such as the FDA’s PCCP. This distinction underscores the evolving tension between innovation and safety in AI-driven kidney care.

One key challenge is the distinction between locked and adaptive AI algorithms, which shapes regulatory pathways and oversight requirements. Locked AI systems employ fixed logic that does not change after deployment, making them predictable and easier to approve under traditional FDA software as a medical device (SaMD) pathways. In contrast, adaptive AI systems continuously learn from new data, update internal logic, and pose complex regulatory challenges due to their evolving behavior. These adaptive systems often require oversight under the FDA’s proposed PCCP, which anticipates future algorithm modifications while ensuring ongoing safety and efficacy. Figure 10 illustrates this regulatory dichotomy and the broader shift toward personalization, highlighting the tradeoff between algorithm stability and adaptability.

A significant regulatory expectation is the demonstration of robust quality-control processes and reproducible, standardized workflows. Variations in data acquisition, hardware and local practices further complicate the regulatory landscape, demanding that developers proactively address these variables to secure approval and ensure patient safety [157]. Simultaneously, privacy regulations impose constraints on data access and multi-institutional collaboration, both of which are critical to assembling diverse representative datasets. Compliance with frameworks such as the Health Insurance Portability and Accountability Act (HIPAA) in the USA [158], the General Data Protection Regulation (GDPR) in the EU [159] and other regional protection laws further complicate data sharing and cross-border AI research and deployment. These constraints have spurred interest in federated learning, synthetic data generation and other privacy-preserving AI techniques as viable strategies for regulatory-compliant innovation in this space [105].

### Clinical integration and workflow

Effective clinical integration remains a central challenge in advancing the adoption of AI tools in nephrology. Similar to traditional CDS systems, AI applications require deliberate embedding within EHR workflows, supported by appropriate governance, oversight and clinician engagement [97]. However, AI systems often introduce additional complexity, including risks of automation bias, increased medico-legal uncertainty and the need for ongoing performance monitoring, which necessitate institutional readiness beyond that required for conventional CDS tools [97, 104].

From a practical standpoint, AI tools are most effective when deployed in clearly defined, high-friction clinical workflows, such as early AKI risk stratification in high-acuity settings, dialysis prescription optimization and post-transplant risk surveillance integrating longitudinal data [1]. In contrast, tools that duplicate routine clinical reasoning or generate non-actionable alerts offer limited benefit. Successful implementation should therefore prioritize narrow use cases, transparent outputs and human-in-the-loop oversight to ensure augmentation of, rather than replacement for, clinical judgment [1].

Key principles include:

- Prioritizing workflow integration: AI solutions must minimize disruption, and those targeting decision-making, should be aligned with the five rights of CDS to provide (i) the right information, (ii) to the right user, (iii) in the right format, (iv) via the right channel and (v) at the right time in the clinicians’ workflow [99]. Embedding into clinical workflow must consider trust, interpretability and clinician incentives to avoid “alert fatigue” and maximize uptake [160]
- Implementation science investigations: successful workflow integration will demand a better understanding of factors that facilitate or hamper the adoption of AI-based systems in nephrology care, which will demand rigorous implementation science studies. Frameworks such as the Consolidated Framework for Implementation Research (CFIR) [98] and Reach Effectiveness Adoption Implementation Maintenance (RE-AIM) [106] can guide the systematic identification of barriers and enablers for adoption and sustainment
- Emphasizing external validation: models should be validated on independent, diverse datasets to ensure generalizability across different populations and care settings [96]. Performance often degrades when applied to new settings, necessitating context-specific validation and adaptation strategies [54]
- Ensuring transparency and simplicity: clinicians must be able to understand model outputs sufficiently to exercise informed professional judgment. “Black box” solutions erode trust and hinder adoption [101]. Explainable AI methods and provider-facing summaries are critical to fostering confidence in model recommendations [10]

The human-in-the-loop paradigm is essential, ensuring that AI augments rather than replaces clinical expertise, supporting decision-making without undermining clinician autonomy.

### Education and training

Despite growing enthusiasm for AI’s potential, there is a significant gap in AI literacy among nephrology professionals. A multicenter survey of nephrology fellows revealed that while 76% perceive AI as moderately to highly relevant to their specialty, an equivalent proportion reported minimal or no clinical experience using AI tools [100]. Importantly, over three-quarters expressed strong interest in formal AI training, identifying interactive workshops as their preferred educational format. This mismatch between interest and training highlights a pressing need for nephrology education programs to incorporate AI-specific competencies, including clinical interpretation, bias awareness and regulatory literacy.

Recent evidence supports the utility of LLM-based tools in nephrology education. The ECOSBot study [161], validated a generative AI platform using GPT-4o to simulate Objective Structured Clinical Examinations (OSCE) stations across five medical schools. ECOSBot demonstrated excellent clinical fidelity, strong alignment with faculty ratings on diagnostic reasoning, and high student satisfaction, offering a scalable solution to augment traditional nephrology training. This underscores the potential role of LLMs in addressing resource limitations and standardizing diagnostic reasoning education in nephrology.

This underscores the urgency of integrating AI literacy into nephrology education, spanning:

- Fundamental principles of AI and ML
- Practical skills in interpreting AI outputs
- Understanding limitations, bias and appropriate use cases
- Regulatory and ethical considerations

Embedding AI content into fellowship training, continuing medical education (CME) modules and board preparation courses may empower clinicians to evaluate and apply AI more confidently in practice. Equipping nephrologists with these competencies is essential for the safe, effective and confident deployment of AI technologies in clinical practice.

### Performance metrics and validation

AI applications across nephrology demonstrate promising technical performance across multiple domains, with most studies reporting moderate to high discrimination metrics. However, interpretation of these metrics requires careful attention to event prevalence, calibration and validation context.

- AKI prediction: models frequently achieve AUROC values between 0.75 and 0.90 [162]. Given the overall low incidence of AKI events, especially in low-risk settings, AUROC alone may overestimate practical performance, underscoring the importance of complementary metrics such as positive predictive value, calibration and decision thresholds when evaluating model validity. Models incorporating multimodal inputs highlight the need for consistent data availability and standardized input definitions when assessing reproducibility across settings [54, 163–165]
- CKD progression: predictive models demonstrate comparable discrimination but benefit from higher event rates and longer prediction horizons, allowing more stable estimation of calibration and risk strata across populations [166]
- Dialysis optimization: AI tools have demonstrated both predictive accuracy and measurable effects on selected outcomes. For these applications, external validation across dialysis centers remains essential to confirm reproducibility and transportability of reported performance

At a systems level, effective validation for clinical deployment requires more than technical performance assessment. Key requirements include multicenter external validation, prospective evaluation of workflow integration, and demonstration of impact on clinician behavior, care processes and patient-centered outcomes. Models must also support recalibration across populations, transparent performance reporting and governance mechanisms for ongoing bias surveillance and performance monitoring over time. Accordingly, successful clinical translation should be anchored by:

- Rigorous multi-site external validation across diverse practice environments
- Prospective evidence of improvements in care processes, safety, efficiency and cost-effectiveness
- Defined performance thresholds validated by domain experts
- Continuous post-deployment monitoring for performance drift and fairness

Ultimately, performance evaluation in nephrology AI must extend beyond discrimination metrics to include reproducibility, calibration stability and transportability across populations and care settings. Table 3 summarizes these multifaceted challenges and potential solutions.

**Table 3:** Key challenges and solutions for AI in nephrology.

Domain	Key challenges	Solutions
Data quality and bias	- Dataset bias across demographics and care settings	- Curate diverse, multicenter datasets
	- Scarcity of data in rare kidney diseases	- Conduct routine bias audits
	- Variability in imaging and pathology protocols	- Standardize data acquisition and preprocessing
Regulatory and privacy	- Complex and evolving approval pathways-	- Engage regulators early in development
	- Privacy regulations limiting data sharing	- Apply privacy-preserving technologies (e.g. federated learning, differential privacy)
Clinical integration	- Limited translation of AI models into routine workflows	- Use implementation science to enable seamless EHR integration
	- Workflow disruption (e.g. alert fatigue)	- Incorporate explainable AI tools
	- Mistrust of “black box” models	- Maintain human-in-the-loop oversight
Education and training	Low AI literacy among nephrologists and trainees	- Integrate formal AI curricula
		- Offer hands-on workshops and case-based training
Performance and validation	- High technical accuracy but low clinical utility	- Conduct external, multi-site validation
	- Poor generalizability to new populations or settings	- Focus on patient-centered outcomes
		- Implement continuous post-deployment monitoring

## CHALLENGES, OPPORTUNITIES AND FUTURE DIRECTIONS FOR AI INTEGRATION IN NEPHROLOGY

- Human and clinical integration barriers: clinician resistance to adopting AI due to trust and medicolegal liability concerns remains a formidable obstacle, particularly when systems are perceived as opaque or misaligned with clinical intuition. Disruption of existing workflows, limited interpretability of complex model outputs, and inadequate user interface design further impede adoption. Addressing these issues will require explainable AI approaches, intuitive interfaces and alignment of AI outputs with real-world clinical workflows to preserve interdisciplinary trust and foster sustained use.
- Data limitations and generalizability: the scarcity of large, high-quality, diverse and well-annotated datasets in nephrology limits generalizability and amplifies bias, particularly in rare kidney diseases, pediatric populations and underrepresented groups. Understudied areas such as glomerular diseases and transplant rejection remain particularly vulnerable to poor performance due to insufficient representation in training datasets. Coordinated, multicenter collaborations, harmonized data repositories and rigorous external validation frameworks are essential to ensure equitable, reproducible performance across diverse clinical settings.
- Cost-effectiveness and reimbursement: despite early studies suggesting potential cost savings from improved efficiency and outcomes, few AI-enabled nephrology tools have undergone formal economic evaluation. Pathways for reimbursement and payer incentives remain unclear. Future work must incorporate cost-effectiveness analyses, budget impact modeling and health economic endpoints into AI implementation trials to ensure financial sustainability and real-world adoption.
- Pediatric nephrology applications: AI research in pediatric AKI, CKD and transplantation remains sparse. Barriers include small sample sizes, developmental heterogeneity and limited pediatric-specific datasets. These challenges also present opportunities to leverage AI in low-resource or low-volume pediatric settings to improve access to specialized care, provided that contextual validation and fairness safeguards are in place.
- Workforce education and cultural readiness: low AI literacy among nephrology trainees and practicing clinicians hinders meaningful adoption. Integrating formal AI training into fellowship curricula, CME modules and multidisciplinary workshops can cultivate clinical AI champions who serve as implementation stewards. Cultural readiness, which includes promoting acceptance, trust and informed engagement, is as critical as technical readiness.
- Innovation and long-term opportunities: the future of AI in nephrology will be shaped by its capacity to unify multimodal datasets, including EHR data, device data, imaging, digital pathology and multi-omics, into biologically informed and clinically actionable models. Reinforcement learning and digital twin technologies offer potential for dynamic and personalized decision-making in areas such as immunosuppression titration, dialysis prescription and antihypertensive management. Generative AI applications in documentation, patient education and clinical triage further expand the scope of innovation.
- Regulatory and governance evolution: regulatory frameworks must evolve to address both static and adaptive AI models, incorporating standardized performance and fairness benchmarks, predetermined change control plans, and clear liability allocation between developers, institutions and clinicians. Post-market surveillance, real-time auditing, and the formation of AI stewardship teams within health systems will be essential to safeguard safety, equity and trust over time.

## LIMITATIONS

Several limitations should be acknowledged. First, this review was not designed as a formal systematic review or meta-analysis, and therefore does not claim exhaustive coverage of all published AI models in nephrology. Instead, the synthesis reflects a state-of-the-art, expert-informed perspective developed through structured literature review and collaborative appraisal by leaders in nephrology, critical care, informatics and AI. While this approach prioritizes clinical relevance, implementation readiness and translational insight, it may introduce selection bias and limits quantitative comparisons across studies. These limitations underscore the need for future systematic reviews and prospective studies to complement expert-driven frameworks for AI integration in kidney care.

## FUTURE DIRECTIONS AND METHODOLOGICAL PRIORITIES

Building on these limitations, several foundational methodological challenges warrant focused attention. Overfitting remains a central concern in nephrology, where small sample sizes, high-dimensional data and disease heterogeneity are common. Models derived from narrowly defined or single-center cohorts may demonstrate strong internal performance yet fail to generalize to broader or more diverse populations, a risk that is particularly pronounced in rare kidney diseases, renal pathology and transplantation. Transparent reporting of model complexity, feature selection and validation strategies is therefore essential to support reproducibility and interpretability.

A related challenge is the distinction between internal validation and robust external or real-world validation. While cross-validation and bootstrapping are necessary during development, they do not substitute for independent testing across institutions, care settings and patient populations, or for prospective evaluation of workflow integration and downstream clinical or system-level outcomes. In parallel, inconsistent use of terminology across AI studies in nephrology complicates comparison and synthesis, particularly when terms such as “validation” or “deployment” are applied without explicit definition. Addressing these issues will require sustained emphasis on standardized terminology, multi-site external validation and prospective assessment of clinical impact to advance AI in nephrology beyond proof-of-concept toward reliable, equitable and clinically meaningful applications.

## CONCLUSION

AI has the potential to fundamentally reshape nephrology by enhancing early disease detection, refining risk stratification and enabling more personalized and anticipatory care across the kidney disease continuum. However, successful translation from innovation to impact hinges on a non-negotiable prerequisite: rigorous external validation coupled with deliberate integration into real-world clinical workflows. Models that demonstrate strong retrospective performance but lack transportability, interpretability or operational alignment risk limited adoption, unintended harm and erosion of clinician trust. As AI systems increasingly influence diagnostic reasoning, therapeutic decisions and resource allocation, nephrologists must assume an active stewardship role by guiding model development, demanding transparent validation, monitoring performance, implementation measures and equity after deployment, and ensuring that AI augments rather than displaces clinical judgment. The future of nephrology is not defined by whether AI is adopted, but by how responsibly it is governed, implemented and sustained to advance patient-centered, equitable and value-based kidney care.

## Data Availability

No new data were generated or analyzed in support of this research.
